# Concurrent manifestations of Horner’s syndrome and esophageal metastasis of breast cancer: case report of a young woman after a period of non-adherence to treatment: a case report

**DOI:** 10.1186/s13256-021-02688-7

**Published:** 2021-04-09

**Authors:** Sumadi Lukman Anwar, Widya Surya Avanti, Lina Choridah, Ery Kus Dwianingsih, Herjuna Hardiyanto, Teguh Aryandono

**Affiliations:** 1 Division of Surgical Oncology-Department of Surgery, Dr. Sardjito Hospital/Faculty of Medicine, Public Health, and Nursing, Universitas Gadjah Mada, Jl Kesehatan No. 1, Yogyakarta, 55281 Indonesia; 2 Department of Radiology, Dr. Sardjito Hospital / Faculty of Medicine, Public Health, and Nursing, Universitas Gadjah Mada, Yogyakarta, 55281 Indonesia; 3 Department of Anatomical Pathology, Dr. Sardjito Hospital/Faculty of Medicine, Public Health, and Nursing, Universitas Gadjah Mada, Yogyakarta, 55281 Indonesia

**Keywords:** Esophageal metastasis, Horner’s syndrome, Breast cancer, Diagnosis, Low adherence

## Abstract

**Background:**

Esophageal involvement and Horner’s syndrome are rare manifestations of breast cancer distant metastases that can pose a significant challenge in diagnosis and treatment. In addition to the more aggressive behavior of breast cancer diagnosed in young women, non-adherence to treatment is associated with increased risk of distant metastasis.

**Case presentation:**

A 36-year-old Javanese woman presented to our institution with dysphagia, hoarseness, and frequent hiccups. In the 6 weeks prior to the current admission, the patient also reported tingling in the neck and shoulder, anhidrosis in the left hemifacial region, and drooping of the upper left eyelid. She was previously managed as tuberculoid laryngitis. Plain X-rays showed burst fractures of the cervical vertebrae and slight pleural effusion. Laryngoscopy revealed bowing of the vocal cords and liquid residue in the vallecula that was reduced upon chin tuck. Esophageal metastasis was confirmed with endoscopy showing thickening of the wall and positive cytology swab with ductal malignant cells. The patient had a history of breast cancer with a period of loss to follow-up of 4 years.

**Conclusions:**

Physicians should consider potential distant metastasis of breast cancer to the esophagus and sympathetic nervous system of the neck particularly in a high-risk woman with presentation of dysphagia and manifestations of Horner’s syndrome.

## Background

Approximately 30% of patients with breast cancer will eventually develop distant metastasis including those who are diagnosed in early stages [1]. Patterns of locoregional recurrence and distant metastasis in patients with breast cancer vary depending on the tumor size, lymph node infiltration, histological subtype, differentiation grades, status of resection margin, immune response, lymphovascular invasion, and intrinsic molecular subtypes [2]. Also, social determinants causing non-adherence in breast cancer treatment have been associated with a higher risk of distant spread and poor functional well-being [3].

The primary sites of breast cancer recurrence are locoregional which include the contralateral breast, lymph nodes, bones, viscera, lungs, liver, and brain [2]. Distant metastasis of breast cancer to the esophagus is very uncommon, accounting for less than 0.5% [4]. In addition, the manifestations of Horner’s syndrome due to disruption of sympathetic nerves in the neck in patients with breast cancer are rarely suspected in most clinics [5]. The infrequent presentation leads to a diagnostic challenge and is often associated with delayed management affecting the patient’s overall survival and quality of life. We report dual presentations of esophageal infiltration and Horner’s syndrome as clinical manifestations of breast cancer metastases that have not been previously reported.

## Case report

A 36-year-old Javanese woman presented to our institution with hoarseness, frequent hiccups, and difficulty in swallowing. She complained of tingling in the neck and shoulder, anhidrosis in the left side of the head, and drooping of the upper left eyelid for the past 6 weeks. The patient had been diagnosed with tuberculosis laryngitis 6 months before the current visit according to positive interferon-gamma release assay (IGRA), although with negative sputum tests and negative biopsy. She had received a combination of tuberculosis drugs for 6 months. The patient had a previous history of invasive ductal carcinoma of the left breast 4 years before the current admission.

**Fig. 1:**
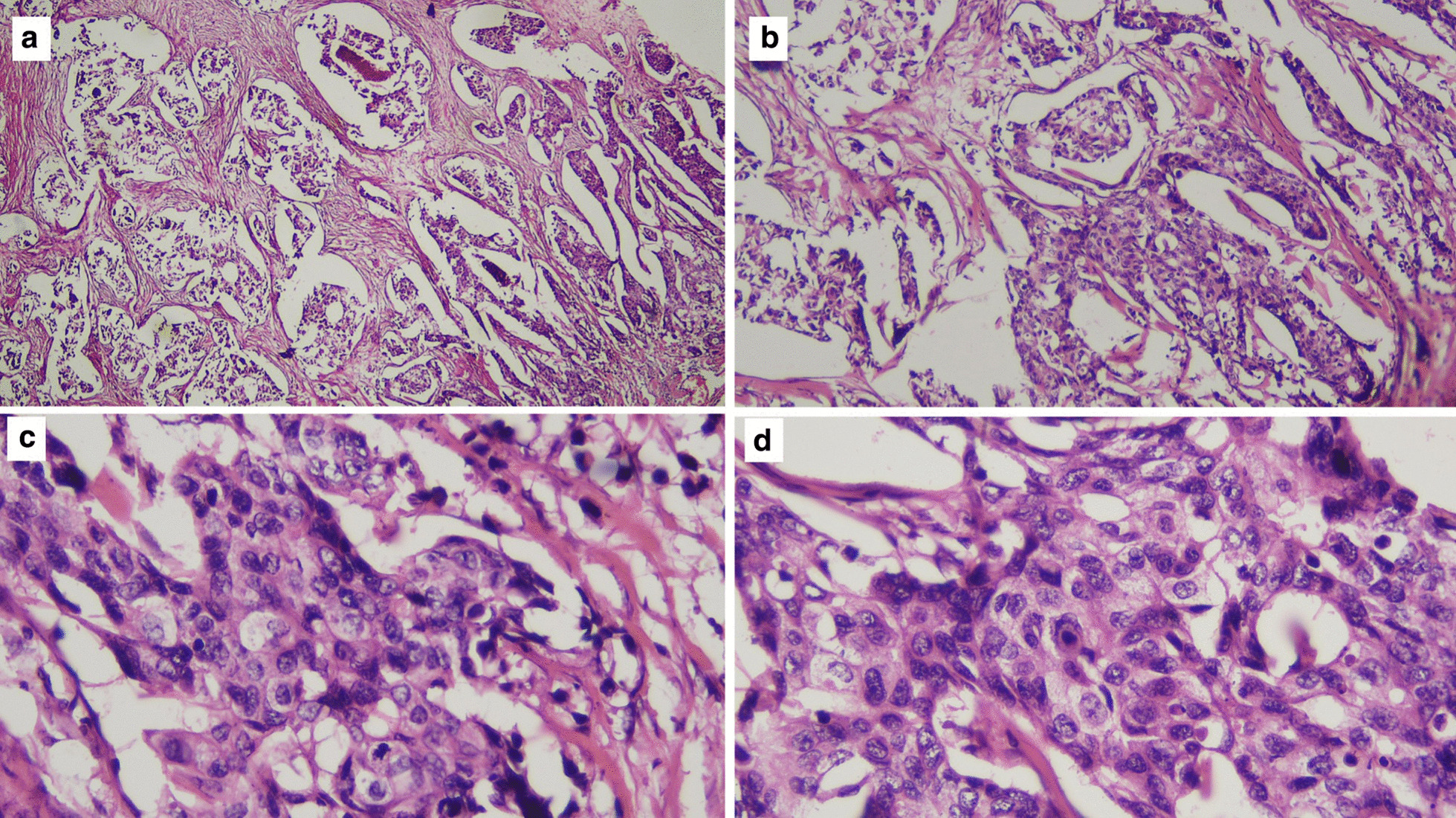
Infiltrative ductal carcinoma with some inflammatory cells in the stroma, which was diagnosed in a 36-year-old woman, showing some characteristics of aggressive behavior. **a**, **b** Tumor nests arranged in solid fashion, indicating poor differentiation (according to Nottingham modified Scarff–Bloom–Richardson classification) and infiltrating surrounding fibrous tissue (×10). **c**, **d** (×40) Tumor cells are large, with vacuolated cytoplasm. Nuclei are round to oval, and some with prominent nucleoli

**Fig. 2:**
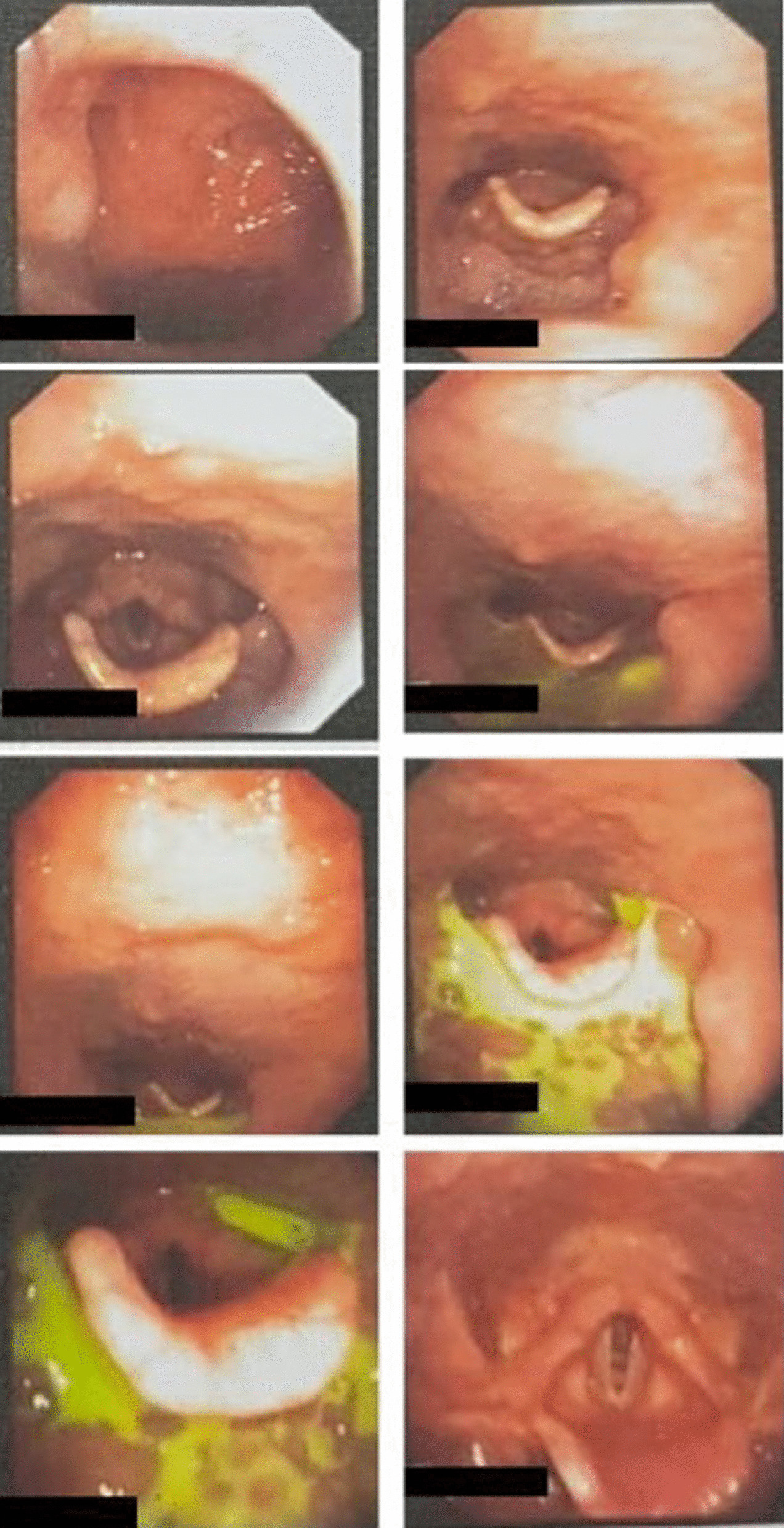
Successive laryngoscopy images showed bowing of vocal cords, a residue of liquid and thick liquid in epiglottic vallecula indicating the dysphagia was caused by pathology in esophageal phase. In addition, the liquid residue was improved upon the chin tuck procedure

**Fig. 3:**
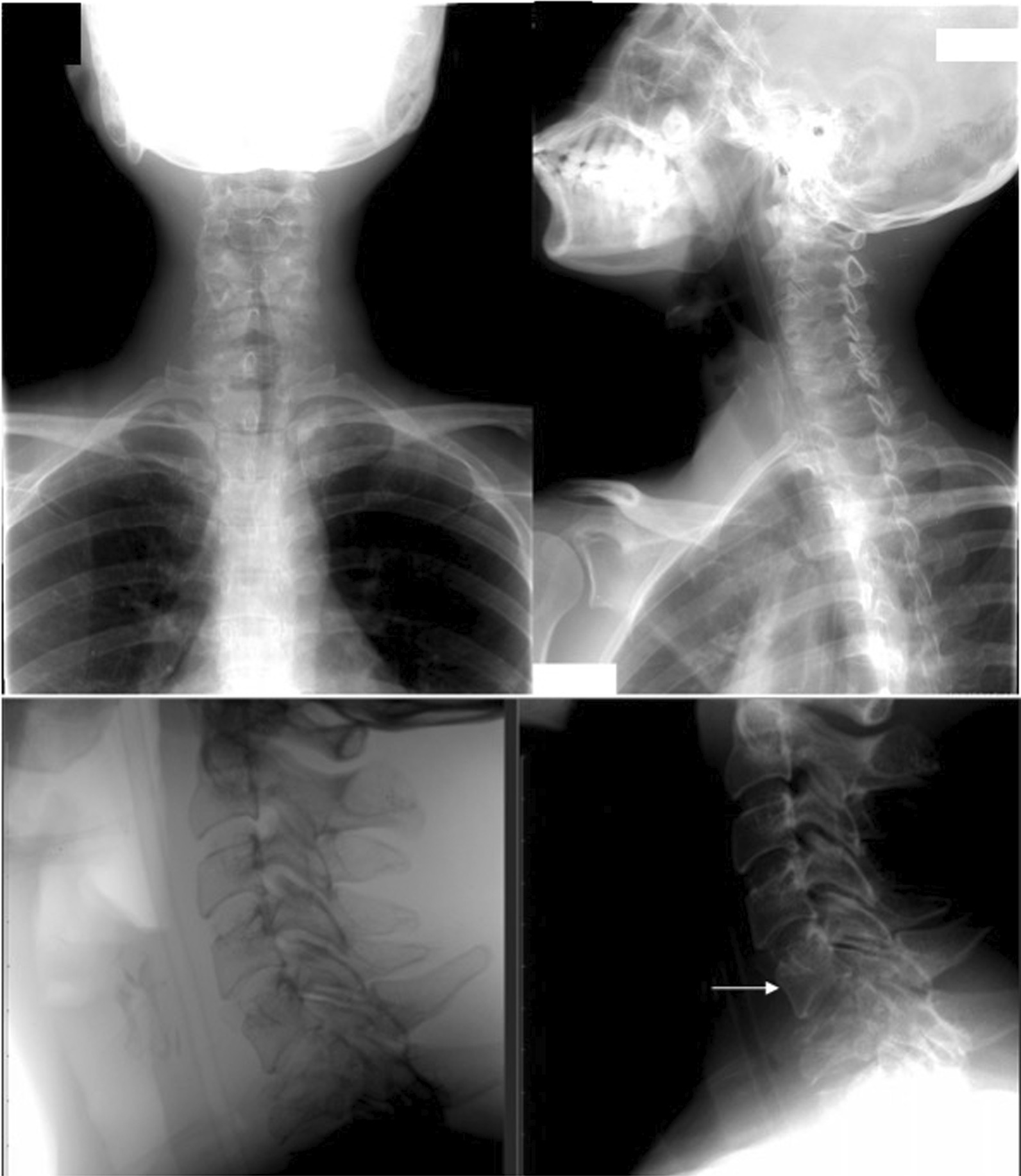
Plain cervical X-ray. The arrow shows burst fracture of the 6th cervical vertebra

**Fig. 4:**
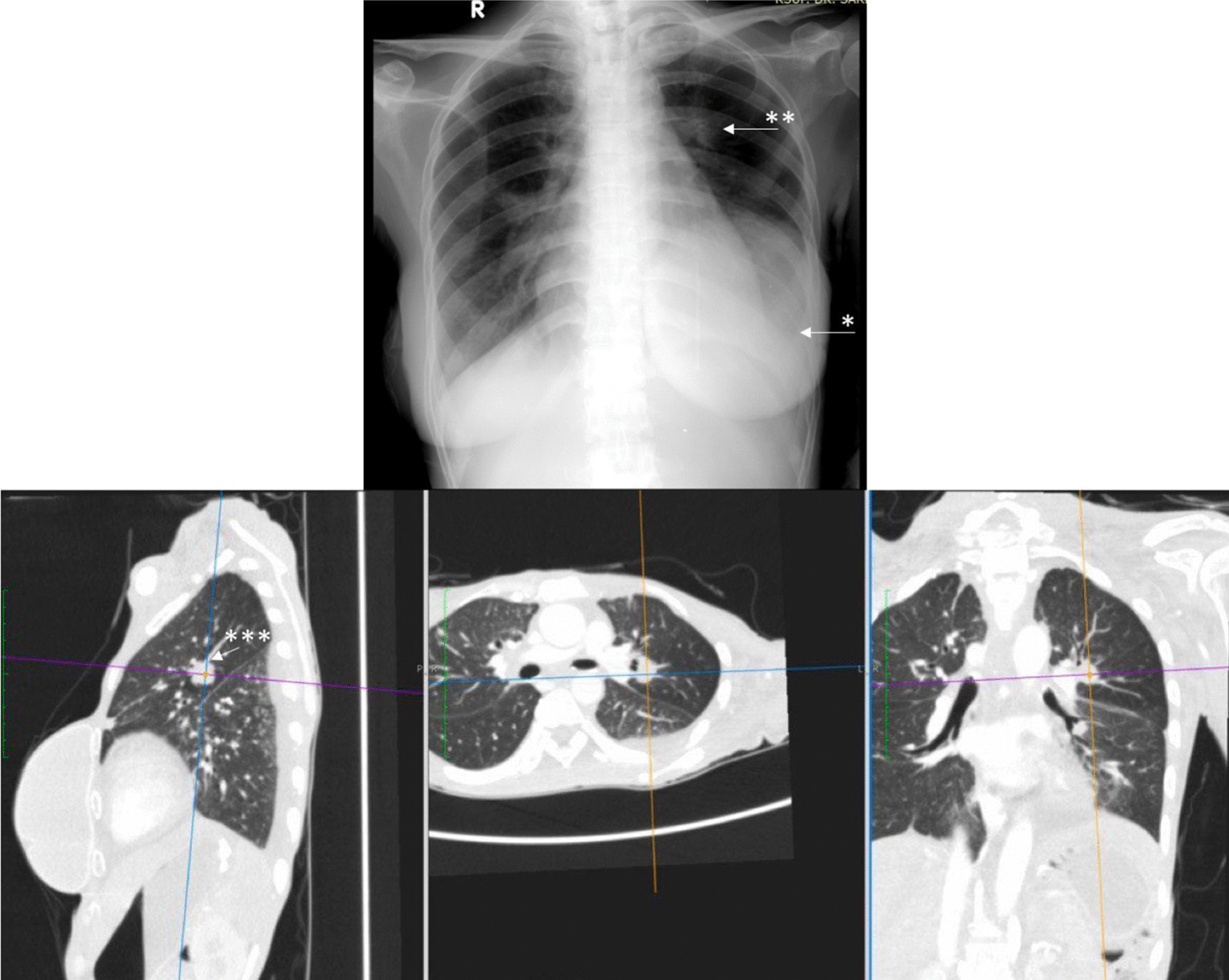
Thoracic X-ray and computed tomography (CT) scan indicate disease spreading into the left pleura and lung. Plain X-ray shows left pleural effusion (*) and nodular lung lesion (**) that were confirmed with CT scan (***)

**Fig. 5:**
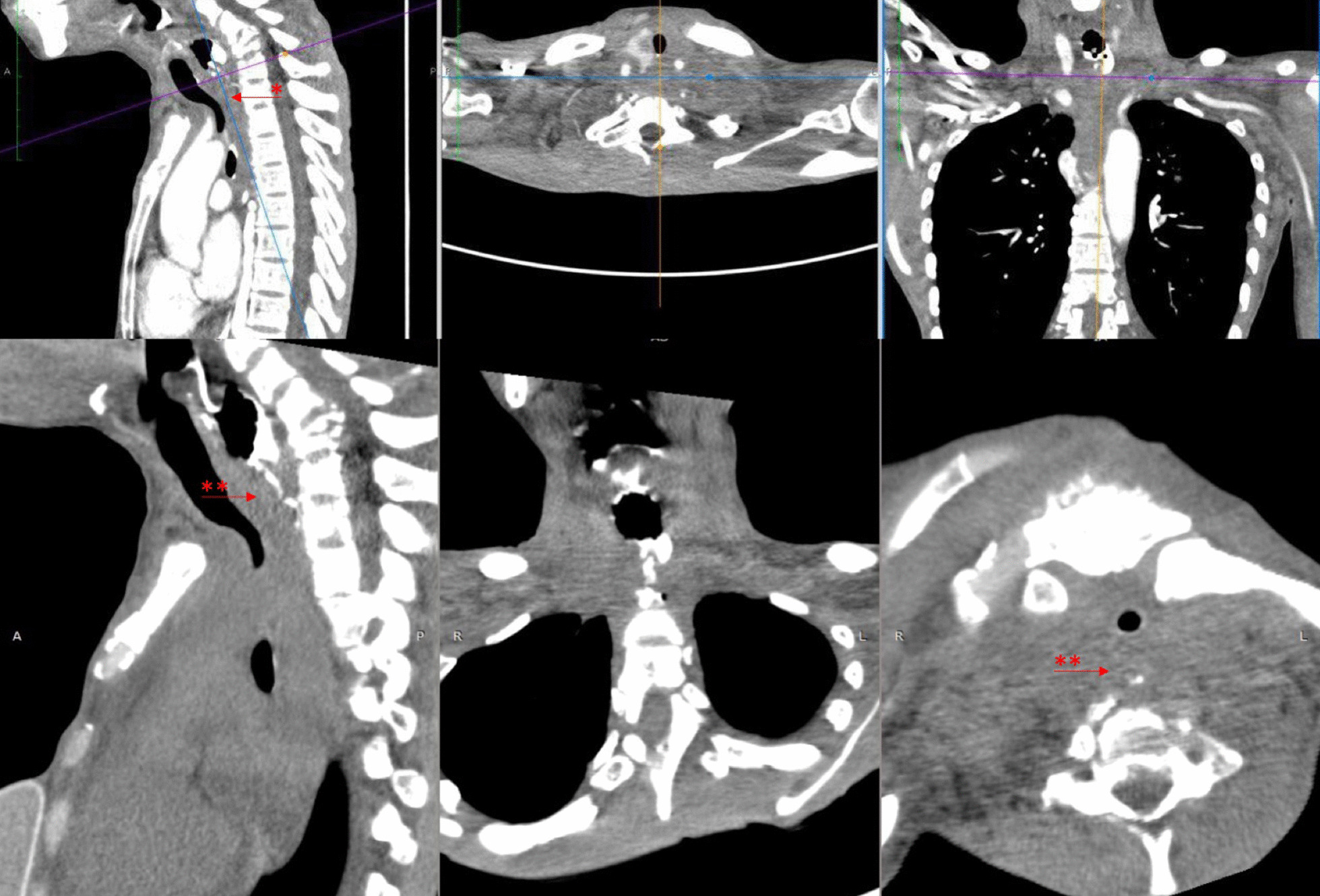
Neck computed tomography scan with oral contrast shows filling defect in the cervical part of the esophagus (in the projection of fifth cervical to first thoracic vertebrae) (*). The amorphous lesion with irregular border appears to compress the esophageal lumen (**)

**Fig. 6:**
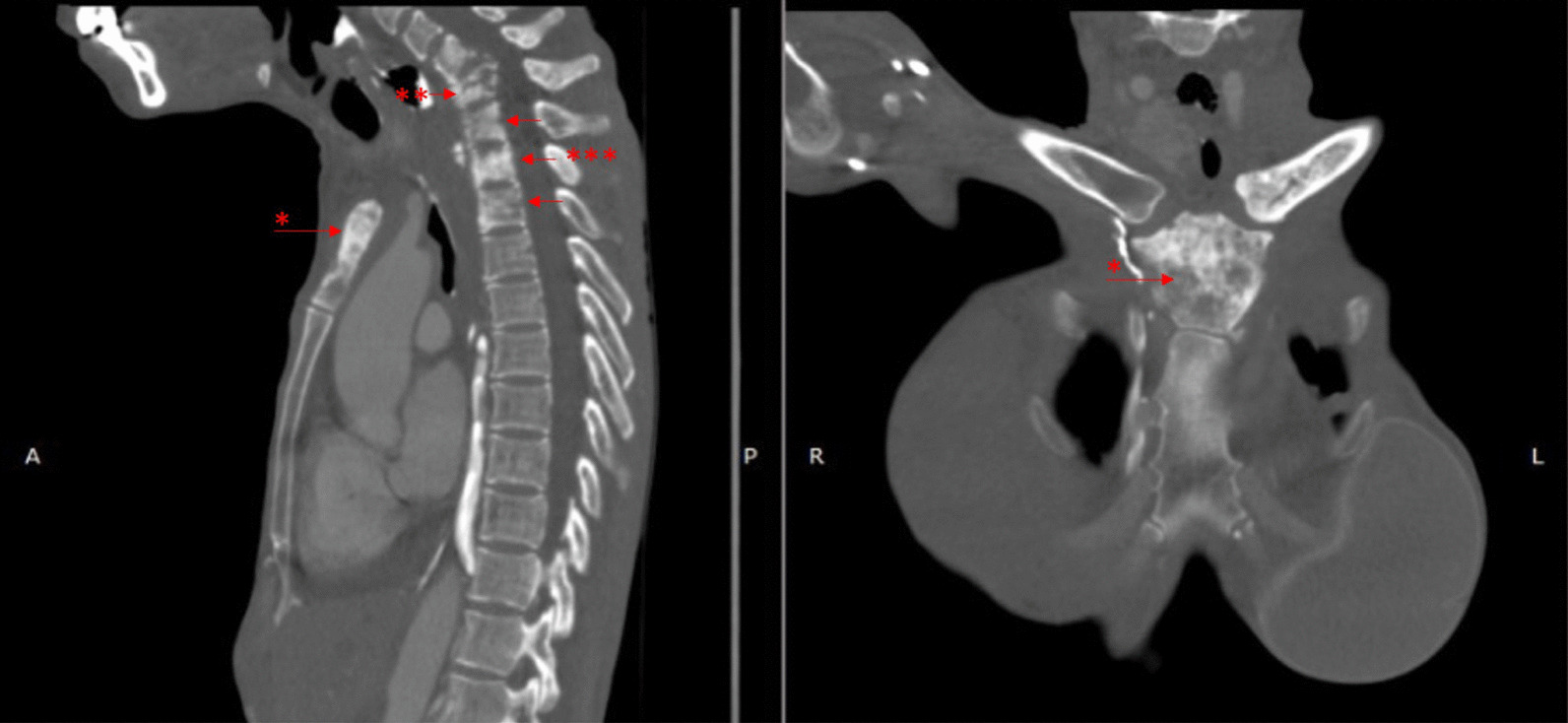
Lytic lesions and bone destruction are found in the manubrium of the sternum (*), burst fracture in the sixth cervical vertebra (**), and lytic lesions in the seventh cervical and first and second thoracic vertebrae (***)

The diagnosis of breast cancer was confirmed at the age of 32 years after a 3-year period of delayed diagnosis because of the patient’s preference to receive traditional (alternative) medicine. At the time of diagnosis, the patient was an unmarried woman but she planned to have a family soon after breast cancer therapy. After a definitive diagnosis of breast cancer was established, the patient had a nipple and skin-sparing mastectomy with immediate reconstruction using a breast implant. Pathological evaluation of the specimen from the mastectomy revealed an invasive ductal carcinoma (Fig. 1) with negative expression of estrogen receptors (ER) and progesterone receptors (PR) and positive expression of HER2 receptors. The primary specimen measured 3 × 5.5 × 3 cm (pT3), with negative axillary lymph node (pN0) and without distant metastasis (M0). The tumor was poorly differentiated according to the Nottingham modified Scarff–Bloom–Richardson (SBR) classification. However, the patient had not continued further medical treatment for the breast cancer until the most recent presentation of hoarseness and difficulty in swallowing (43 months after the initial diagnosis). The hoarseness was previously suspected to be due to tuberculoid laryngitis according to IGRA, although with negative acid-fast bacilli sputum tests and negative biopsy. The dysphagia was managed with nasogastric tube insertion. Anti-tuberculosis treatment for the past 6 months did not improve the patient’s complaints of hoarseness and dysphagia. In addition, she complained of left-side facial anhidrosis, left ptosis, and myosis of the left eye, and accordingly a diagnosis of Horner’s syndrome was established. Laryngoscopy revealed bowing of the vocal cords, a residue of liquid and thick liquid in the epiglottic vallecula, with a slight improvement of food residue after chin tuck (Fig. 2). In addition, the patient complained of tingling in the neck accompanied by a recurrent pain in the face. Cervical X-ray of the neck revealed a burst fracture of the sixth cervical vertebra (Fig. 3). Chest X-ray indicated the disease was spreading into the left pleura and lung, which was confirmed by computed tomography (CT) scan (Fig. 4). In addition, neck CT scan showed an intraluminal esophageal mass at the level of the sixth cervical to the first thoracic vertebrae (Fig. 5) and lytic destruction of the manubrium of the sternum, sixth and seventh cervical vertebrae, and first to third thoracic vertebrae (Fig. 6). Endoscopy confirmed the thickening of the esophageal wall, and cytology swab showed ductal malignant cells suggesting distant metastasis of breast cancer into the intraluminal esophagus. A gastrostomy tube was inserted to maintain caloric and nutrition intake. The patient was then treated with capecitabine 1250 mg/m^2^ twice daily for 14 days, with 6 mg of trastuzumab and 4 mg of zoledronic acid in a 3-week cycle for eight cycles. After the treatment, the patient showed improvements in ingestion and swallowing and was able to drink and take soft food, although the signs of Horner’s syndrome did not improve.

## Discussion

Breast cancer is ranked the most frequent cancer among women worldwide [6]. Breast cancer-associated mortality has gradually declined during the past 25 years due to improvements in surgery and adjuvant oncologic treatment as well as early detection and screening [7]. However, breast cancer mortality rates are higher in low- and middle-income countries (LMICs) [7], including Indonesia. Late presentations with more advanced cases due to delayed diagnosis and lower adherence to treatment are associated with a higher risk of locoregional and distant spread [8]. Distant metastasis of breast cancer is commonly found in the bones, liver, lungs, and brain, whereas neck and esophageal metastasis is very rare (accounting for only around 0.5%) [4]. Lobular cancer is a histological type of breast cancer that frequently metastasizes into the gastrointestinal system, particularly to the stomach and colon [4, 9]. We report a rare case of metastatic ductal carcinoma of the breast in a young woman with Horner’s syndrome and infiltration to the esophagus.

Esophageal metastasis from breast cancer has been reported in around 39 cases, mostly in postmenopausal women who were detected in a latent interval of more than 7 years after the initial diagnosis, as summarized by Miyake *et al*. [4]. Rampado *et al*. reported 25 cases of “breast-esophagus syndrome” in patients with a median age of 58 years who presented with esophageal symptoms after a median follow-up of 10 years [10]. No report has previously described esophageal metastasis in a young woman under 40 years of age with accompanying signs of Horner’s syndrome. The predilection for esophageal metastasis is in the middle to distal third, and the latent interval between diagnosis and esophageal spread ranges between 2 and 24 years [4, 10]. Dysphagia due to compression or stenosis of the esophagus, hoarseness, and slimming are the most common symptoms of esophageal spread, although these symptoms are present in only 30% of patients [4, 10]. Therefore, the diagnosis of esophageal spread is challenging and requires further tests to differentiate from primary esophageal tumors, benign strictures, and chronic inflammation. In our case, we initially suspected tuberculous laryngitis according to the positive IGRA and relatively high incidence of local tuberculosis, although the patient’s symptoms were not alleviated after treatment for 6 months.

Horner’s syndrome, or oculosympathetic paresis, is characterized by ipsilateral miosis, partial ptosis, and anhidrosis due to disruption of the sympathetic arc nerve passing from the hypothalamus through a long pathway to the eye [11]. The first order of the central neuron arises from the hypothalamus to the brainstem and leaves through the thoracic levels (C8–T2) of the spinal cord [11]. The second neuron (preganglionic) incorporates into the sympathetic trunk crossing the brachial nerve above the apex of the lung to form a plexus with the superior cervical ganglia [11]. The third branch (postganglionic) travels alongside the carotid sheath to enter the eye bulb to enervate the pupil dilator muscle, the lacrimal sac, and superior tarsal Müller muscle [11]. The third postganglionic fibers that regulate facial hidrosis often follow the passage of the external carotid artery; therefore, defects in the postganglionic nerve do not always result in anhidrosis [11]. Disruption of the sympathetic trunk can occur in the central, preganglionic, and postganglionic nerves [11], in which trauma often affects the preganglionic nerve, whereas dysfunction of the postganglionic nerve causing acquired Horner’s syndrome is mostly caused by Pancoast tumors or metastatic cancer. We found four reports describing Horner’s syndrome resulting from distant metastasis of breast cancer. Tjalma *et al*. reported Horner’s syndrome as an ominous sign of metastatic breast cancer in which disruption of the sympathetic trunk was caused by mediastinal lymph node infiltration [12]. In 2007, Kovacic *et al*. described Horner’s syndrome resulting from malignant pleural effusion in metastatic breast cancer [5]. Vitale *et al*. reported Horner’s syndrome resulting from metastases to left-lateral cervical and supraclavicular lymph nodes in a 52-year-old woman with breast cancer [13]. Park *et al*. described Horner’s syndrome due to breast cancer spread in the cervicothoracic junction as shown by intense hypermetabolic signals of positron emission tomography (PET) scan [14]. In our case, bone metastasis in the cervical and thoracic vertebrae and in the soft tissues surrounding the esophagus might have caused the manifestations of Horner’s syndrome.

The biological nature of esophageal and neck spread of breast cancer is still under study. It is suggested that the cancer cells travel through intramammary lymphatic vessels into periesophageal lymph nodes through the parasternal lymph nodes and mediastinum, and in some cases might spread into the intramural layer of the esophagus, causing stricture or compression [9]. In the absence of mediastinal and parasternal lymph node involvement, the esophageal spread might be caused by hematogenous metastasis [4]. In our case, esophagoscopy revealed esophagus wall thickening in the distal part with positive ductal carcinoma cells from fine-needle aspiration biopsy (FNAB). One study described the advantages of esophagoscopy-FNAB for confirming esophageal spread because mucosal and submucosal biopsies are often negative for intramural and nodal involvement [15]. Management of esophageal spread from breast cancer varies among cases and is usually formulated according to the severity levels of the esophageal obstruction and other accompanying distant metastatic sites [4]. Although esophagectomy or metallic stents are considered effective management, surgical resection is generally not performed because systemic treatment is preferred in the presence of visceral crisis or distant metastasis [4]. In addition, chemotherapy, hormone therapy, and selective radiotherapy are also effective treatments for esophageal metastasis from breast cancer. After the establishment of esophageal spread from breast cancer, our patient was treated with capecitabine, trastuzumab, and zoledronic acid, resulting in the alleviation of dysphagia symptoms, and she was then able to swallow softened food.

Distant metastasis of breast cancer has typically been reported 8–30 years after primary treatment [1, 2]. Our patient presented with distant spread 4 years following the initial diagnosis and surgery. In addition to the biological nature of more aggressive behavior in breast cancers diagnosed in young women [16], delayed diagnosis might contribute to the elevated risk of distant metastasis in the present case. In addition, the standard adjuvant treatment after surgery was not delivered due to loss of follow-up for 4 years until the presentation of distant metastasis. Our case also reflects the complexity of sociocultural factors in the management of breast cancer in LMICs, in which low cancer awareness, a misconception concerning cancer treatments and cures, and barriers to accessing medical treatment are associated with delayed diagnosis, late-stage presentation, and a higher risk of metastasis [17–20]. Similar factors are associated with low and non-adherence to medical treatment of breast cancer. Therefore, effective communication with patients and their family members should be delivered by health care providers to improve treatment adherence. In addition, public health intervention is needed to prevent and reduce delayed diagnosis and cancer progression after the initial successful treatment.

## Conclusions

The co-occurrence of esophageal spread and Horner’s syndrome in a young woman with breast cancer is very rare and, to our knowledge, has never been reported previously. Early recognition, diagnosis, and treatment workups are very challenging, particularly in a patient with low or non-adherence to cancer treatment. Systemic treatment remains the most preferred option for esophageal metastasis, particularly in a patient with other synchronous metastatic sites.

## Data Availability

All data are available in the hospital’s medical records.
